# Prolonged Sitting, Its Combination With Physical Inactivity and Incidence of Lung Cancer: Prospective Data From the HUNT Study

**DOI:** 10.3389/fonc.2019.00101

**Published:** 2019-02-25

**Authors:** Lin Jiang, Yi-Qian Sun, Ben Michael Brumpton, Arnulf Langhammer, Yue Chen, Tom I. L. Nilsen, Xiao-Mei Mai

**Affiliations:** ^1^Department of Public Health and Nursing, Faculty of Medicine and Health Science, Norwegian University of Science and Technology, Trondheim, Norway; ^2^Department of Clinical and Molecular Medicine, Faculty of Medicine and Health Science, Norwegian University of Science and Technology, Trondheim, Norway; ^3^Department of Thoracic Medicine, St. Olavs Hospital, Trondheim University Hospital, Trondheim, Norway; ^4^K.G. Jebsen Centre for Genetic Epidemiology, Department of Public Health and Nursing, Norwegian University of Science and Technology, Trondheim, Norway; ^5^MRC Integrative Epidemiology Unit, University of Bristol, Bristol, United Kingdom; ^6^School of Epidemiology and Public Health, Faculty of Medicine, University of Ottawa, Ottawa, ON, Canada; ^7^Clinic of Anesthesia and Intensive Care, St. Olavs Hospital, Trondheim University Hospital, Trondheim, Norway

**Keywords:** prolonged sitting, physical inactivity, lung cancer risk, prospective cohort, HUNT study

## Abstract

**Background:** Prolonged sitting as a major sedentary behavior potentially contributes to illness, but its relation with lung cancer risk is unclear. Prolonged sitting can be presented in physically active or inactive individuals. Those who are extendedly seated and also physically inactive may represent the most sedentary people. We therefore aimed to prospectively examine if total sitting time daily itself or in combination with physical activity is associated with lung cancer incidence overall and histologic types.

**Methods:** We included 45,810 cancer-free adults who participated in the second survey of HUNT Study in Norway (1995–97), with a median follow-up of 18.3 years. Total sitting time daily and physical activity were self-reported at baseline. Lung cancer cases were ascertained from the Cancer Registry of Norway. Cox regression was used to estimate hazard ratios (HRs) with 95% confidence intervals (CIs).

**Results:** In total, 549 participants developed lung cancer during the follow-up. Total sitting time daily was not associated with the incidence of lung cancer overall and histologic subtypes. Compared with participants sitting < 8 h daily and being physically active, those sitting ≥8 h daily (prolonged sitting) and being physically inactive had an increased incidence of lung cancer (overall: adjusted HR = 1.44, 95% CI: 1.07–1.94; small cell lung cancer: adjusted HR = 2.58, 95% CI: 1.23–5.41). Prolonged sitting only or physical inactivity only was not associated with the incidence of lung cancer.

**Conclusions:** Our study suggested that prolonged sitting was not independently associated with lung cancer incidence. The combination of prolonged sitting and physical inactivity might increase the risk of lung cancer. However, residual confounding by smoking cannot be excluded completely even though smoking was adjusted for with detailed information.

## Introduction

Lung cancer is one of the most common cancer types with a low survival rate (1). Small cell (SCLC) and non-small cell lung cancer (NSCLC) are the two major histologic types of lung cancer (2). Smoking is the most important risk factor for lung cancer, and less so for NSCLC than SCLC (3, 4). With a declining trend in smoking, other lifestyle factors may become more important for the incidence of lung cancer overall and histologic types. Physical activity or sedentary behavior has been suggested to be associated with the risk of cancer due to several plausible mechanisms including suppressed lipoprotein lipase activity (5, 6) and altered inflammatory pathways by lack of activities (7–9).

The relationship of physical activity with lung cancer risk has been extensively investigated. Recent meta-analysis studies have concluded that physical activity is associated with a reduced risk of lung cancer in smokers (10–12). Nonetheless, potential effects of physical activity and sedentary behavior might tangle in these meta-analysis studies since sedentary behavior was not properly taken into consideration in most of the individual studies.

Sedentary behavior describes a series of human behaviors requiring low energy expenditure in a sitting or reclining posture when awake (13). It is highly prevalent in western countries (14) and may be an independent risk factor for multiple health outcomes, including cancers (15, 16). Previous studies focused either on occupational sitting (17–19) or leisure-time TV watching (20, 21) in relation to lung cancer risk, with inconsistent results. Total sitting time daily is a better marker that reflects a sedentary lifestyle in the workplace, domestic environment and during leisure-time (22). However, there are limited studies on the relationship between total sitting time and lung cancer risk, and among them, one study found the association in a sub-population (23) and two studies did not adjust for physical activity properly due to lack of detailed information (21, 23). Physical inactivity and prolonged sitting are two distinct behaviors. Prolonged sitting can be present in physically active or inactive individuals. Those who are extendedly seated and also physically inactive may represent the most sedentary people. Thus, in the current study we aimed to prospectively examine the relationship between total sitting time daily and lung cancer risk (overall and major histologic types), taking smoking into consideration. We also investigated if different combinations of total sitting time and physical activity were associated with lung cancer incidence.

## Materials and Methods

### Study Design and Population

The HUNT study is a large population-based health study in Norway, which includes more than 97% Caucasian participants and well-represents the Norwegian population. It consists of three consecutive surveys; HUNT1 (1984–1986), HUNT2 (1995-1997), and HUNT3 (2006–2008) (24). At each survey, all adults 20 years or older living in the area of Nord-Trøndelag were invited to complete extensive health and lifestyle questionnaires and undergo a clinical examination (25).

A total of 65,229 adults participated (70% of invited) in HUNT2. Every participant was followed-up from the date of participation in HUNT2 until the date of first diagnosis of lung cancer, the date of death or emigration from Norway or the end of follow-up on December 31, 2014, whichever came first. Diagnosis of lung cancer was obtained from the Cancer Registry of Norway. Information on vital status and emigration was obtained from the Central Population Registry.

Among the 65,229 participants, 59,070 self-reported no cancers at baseline. We included 45,810 cancer-free participants with complete information on total sitting time daily in the main cohort. Additionally, we investigated the combination of total sitting time and physical activity in relation to lung cancer risk in a sub-cohort of 33,793 participants who also provided complete information on physical activity.

The study was approved by the Regional Committee for Medical and Health Research Ethics of South-East Norway. All participants signed informed written consent on participation in HUNT, linkage to previous HUNT surveys and specific registries in accordance with the Declaration of Helsinki.

### Ascertainment of Lung Cancer

By using the unique 11-digit personal identification number, participants' information from HUNT2 was linked to the Cancer Registry of Norway (26). Data from the Cancer Registry of Norway are reasonably accurate, complete (overall completeness 98.8% in 2001–05) and timely (27). The International Classification of Diseases version 10 (ICD-10) codes used for registration of lung cancer are C33-C34 (28). Histologic types were classified according to International Classification of Diseases of Oncology (ICD-O) (29).

### Measurement of Exposures

Time spent sitting daily was measured by the question: “How many hours do you usually spend in the sitting position during a 24-h period? (work, meals, television, car etc.).” The participant reported the total number of hours as a positive integer. We categorized total sitting time daily into three categories (0–4, 5–7, and ≥8 h) based on similar cutoff criteria from former HUNT studies (30, 31) and two meta-analysis studies (32, 33). Occupational activity was used as an alternative marker for a sedentary lifestyle and measured by the question: “How would you describe your work?” Based on four response options, we categorized it into mostly sedentary work, much walking or lifting (two response options collapsed into one category), heavy physical work, and an “unknown” group with missing information.

Leisure-time physical activity was based on the question “How much of your leisure time have you been physically active per week during the last year?” Participants were asked to report average hours of light (no sweating or not being out of breath) and hard physical activity (sweating or out of breath) with the following response options for each intensity; none, <1 h, 1–2 h, and ≥3 h (reported as a positive integer). We classified participants' physical activity level as inactive (no any activity, or ≤2 h light activity only), low (≥3 h light activity only, or ≤2 h light activity and < 1 h hard activity), moderate (≥3 h light activity and < 1 h hard activity, or 1–2 h hard activity regardless of light activity), and high (≥3 h hard activity regardless of light activity). This classification has demonstrated a dose-response relationship with mortality (34). Based on information of total sitting time and physical activity, we defined four combined categories: (1) total sitting time < 8 h daily and physically active; (2) total sitting time < 8 h daily and physically inactive; (3) total sitting time ≥8 h daily and physically active; and (4) total sitting time ≥8 h daily and physically inactive. Physically active referred to physical activity level from low to high. Physically inactive referred to no any activity or ≤2 h light activity only.

### Information on Other Important Baseline Variables

Age, sex, body mass index (BMI), active smoking (status and pack-years), and passive smoking status, alcohol consumption, education, economic difficulties, family history of cancer and chronic bronchitis were included *a priori* as potential confounders. Weight and height in HUNT2 were measured by health professionals at clinical examination. BMI was calculated as weight in kilograms divided by height squared in meter (kg/m^2^) and was grouped into three categories (< 25.0, 25.0–29.9, and ≥30.0 kg/m^2^) according to the recommendations of the World Health Organization (WHO) (35). Active smoking status was classified into never, former, current smokers and further classified based on pack-years (≤10.0, 10.1–20.0, and >20.1). Other variables were categorized as: passive smoking (never, only childhood, only adulthood, and both), alcohol consumption (never, 1–4, and ≥5 times/month), education (< 10, 10–12, and ≥13 years, reported as a positive integer), economic difficulty (During the last year, has it at any time been difficulty to meet the cost of food, transportation, housing and such? yes/no), family history of cancer (Is there any family member such as father, mother, siblings who reported cancer? yes/no), and chronic bronchitis (Have you had a cough with phlegm for periods of at least 3 months during each of the last 2 years? yes/no). All missing information on the aforementioned variables was taken into analysis as an “unknown” category.

### Statistical Analysis

Baseline characteristics of participants were presented by the categories of exposure variables. We used Cox proportional hazard models to examine the associations between total sitting time, and its combinations with physical activty and lung cancer incidence overall and histologic types and presented crude and adjusted hazard ratios (HR) with 95% confidence intervals (CI). Age was used as the time scale, with entry and exit time defined as the subject's age at recruitment and age at lung cancer diagnosis or censoring, respectively. When potential linear association between total sitting time and lung cancer risk was evaluated by likelihood ratio test, non-linearity was suggested (*p* = 0.02 for comparison between total sitting time used as a categorical variable and it used as an ordinal variable). Thus, the three categories (0–4, 5–7, and ≥8 h) of total sitting time were applied in the main analysis. Total sitting time was also categorized into tertiles.

In the sensitivity analysis, we first used occupational inactivity (mostly sedentary work) to test the robustness of our results on the assciation between total sitting time daily and lung cancer risk. Secondly, we performed analysis on the association of the combination groups of total sitting time and physcial activity with lung cancer risk after excluding the first three years of follow-up (*n* = 33,322) to reduce possible reverse causality by existing but undiagnosed lung cancer. In addition, we redefined the combination groups by including low activity into the physical inactivity level and repeated the analysis. Physical inactivity was thus redefined as no activity, any light activity only, or light activity ≤2 h and hard activity < 1 h weekly. In this way, we could test if the original category of the combined sitting time ≥8 h and physical inactivity captured the most sedentary individuals. We further conducted complete case analysis among individuals with complete information on smoking (n = 31,907) to minimize residual confounding from smoking. All statistical analyses were performed with STATA/SE 14.2 (College Station, TX, USA).

## Results

In total, 549 participants developed lung cancer during a median follow-up time of 18.3 years among the 45,810 subjects. Tables 1, 2 describe the distribution of baseline characteristics of participants according to total sitting time and its combination with physical activity levels. Compared to participants sitting < 4 h or between 5 and 7 h daily, participants sitting ≥8 h were more likely to be males, frequent drinkers, have higher education and sedentary work (Table 1). Compared to participants who were sitting < 8 h daily and physically active, people who were sitting ≥8 h daily and physically inactive were older, lower educated, more frequent smokers and sedentary workers. Supplementary Table 1 shows that as compared to the original cancer-free population, participants in the main cohort had a higher percentage of family history of cancer and participants in the sub-cohort were relatively younger.

**Table 1:** Baseline characteristics of participants according to total sitting time, the HUNT Study, 1995–97 (*N* = 45,810).

Total sitting time (hours^*^/day)						
**Variables**	**0–4 *N* = 14,258**		**5–7 *N* = 14,549**		**≥8 *N* = 17,003**	
Age (years)	48.5	16.1	49.1	16.8	47.0	16.3
Body mass index (kg/m^2^)	26.1	4.0	26.3	4.1	26.3	4.0
**SEX**						
Female	8,013	56.2	7,796	53.6	7,977	46.9
Male	6,245	43.8	6,753	46.4	9,026	53.1
**SMOKING STATUS (PACK-YEARS)**						
Never	6,181	43.4	6,075	41.8	7,477	44.0
Former ≤10.0	1,992	14.0	2,055	14.1	2,387	14.0
Former 10.1–20.0	730	5.1	858	5.9	981	5.8
Former >20.1	408	2.9	486	3.3	649	3.8
Current ≤10.0	1,568	11.0	1,551	10.7	1,708	10.1
Current 10.1–20.0	1,327	9.3	1,395	9.6	1,509	8.9
Current >20.1	855	6.0	1,022	7.0	1,237	7.3
Unknown	1,197	8.4	1,107	7.6	1,055	6.2
**PASSIVE SMOKING STATUS**						
Never	2,716	19.1	2,596	17.8	3,190	18.8
Only childhood	3,187	22.4	2,962	20.4	3,888	22.9
Only adulthood	2,364	16.6	2,446	16.8	2,467	14.5
Both childhood and adulthood	5,689	39.9	6,260	43.0	7,152	42.1
Unknown	302	2.1	285	2.0	306	1.8
**PHYSICAL ACTIVITY**						
Inactive	2,891	20.3	3,129	21.5	3,911	23.0
Low	2,467	17.3	2,672	18.4	3,331	19.6
Moderate	3,123	21.9	3,438	23.6	4,499	26.5
High	1,457	10.2	1,302	9.0	1,573	9.3
Unknown	4,320	30.3	4,008	27.6	3,689	21.7
**ALCOHOL CONSUMPTION (TIMES/MONTH)**						
Never	4,994	35.0	4,983	34.3	4,751	27.9
1-4	6,810	47.8	6,946	47.7	8,558	50.3
≥5	1,298	9.1	1,570	10.8	2,785	16.4
Unknown	1,156	8.1	1,050	7.2	909	5.4
EDUCATION (YEARS^*^)						
< 10	5,145	36.1	5,170	35.5	4,063	23.9
10-12	5,281	37.0	5,133	35.3	5,281	31.1
≥13	3,376	23.7	3,783	26.0	7,246	42.6
Unknown	456	3.2	463	3.2	413	2.4
**ECONOMIC DIFFICULTIES**						
Yes	4,102	28.8	3,927	27.0	4,305	25.3
No	8,230	57.7	8,451	58.1	10,617	62.4
Unknown	1,926	13.5	2,171	14.9	2,081	12.2
**FAMILY HISTORY OF CANCER**						
Yes	4,171	29.3	4,382	30.1	4,888	28.8
No	10,087	70.8	10,167	69.9	12,115	70.7
**CHRONIC BRONCHITIS**						
Yes	433	3.0	471	3.2	550	3.2
No	13,561	95.1	13,811	94.9	16,182	95.2
Unknown	264	1.9	267	1.8	271	1.6
**OCCUPATIONAL ACTIVITY**						
Most sedentary work	839	5.9	1,736	11.9	8,706	51.2
Much walking or lifting at work	8,409	59.0	7,678	52.8	4,323	25.4
Heavy physical work	2,226	15.6	1,655	11.4	721	4.3
Unknown	2,784	19.5	3,480	23.9	3,253	19.1

*Continuous variables are presented with mean and standard deviation.Categorical variables are presented with number and column percentage of observations*.

* *Hours of total sitting time daily and years of education were reported as a positive integer*.

**Table 2:** Baseline characteristics of participants according to combined total sitting time and physical activity level, the HUNT Study, 1995–97 (*N* = 33,793).

**Total sitting time and physical activity level**								
**Variables**	**Sitting <8 h &Physically active *N* = 14,459**		**Sitting <8 h & Physically inactive *N* = 6,020**		**Sitting ≥8 h & Physically active *N* = 9,403**		**Sitting ≥8 h& Physically inactive *N* = 3,911**	
Age (years)	44.2	15.3	49.4	16.5	42.2	14.0	49.8	17.2
Body mass index (kg/m^2^)	25.8	3.8	26.7	4.4	25.8	3.7	26.8	4.5
**SEX**								
Female	7,215	49.9	3,616	60.1	4,030	42.9	2,018	51.6
Male	7,244	50.1	2,404	39.9	5,373	57.1	1,893	48.4
**SMOKING STATUS (PACK-YEARS)**								
Never	6,636	45.9	2,407	40.0	4,564	48.5	1,514	38.7
Former ≤10.0	2,283	15.8	829	13.8	1,469	15.6	517	13.2
Former 10.1–20.0	744	5.2	340	5.7	482	5.1	248	6.3
Former >20.1	371	2.8	213	3.5	275	2.9	179	4.6
Current ≤10.0	1,690	11.7	710	11.8	977	10.4	437	11.2
Current 10.1–20.0	1,158	8.0	671	11.2	713	7.6	402	10.3
Current >20.1	723	5.0	464	7.7	497	5.3	394	10.1
Unknown	854	5.9	386	6.4	426	4.5	220	5.6
**PASSIVE SMOKING STATUS**								
Never	2,844	19.7	1,016	16.9	1,890	20.1	667	17.1
Only childhood	3,600	24.9	1,208	20.1	2,532	26.9	747	19.1
Only adulthood	2,056	14.2	1,032	17.1	1,125	12.0	660	16.9
Both	5,775	39.9	2,692	44.7	3,745	39.8	1,787	45.7
Unknown	184	1.3	72	1.2	111	1.2	50	1.3
**ALCOHOL CONSUMPTION (TIMES/MONTH)**								
Never	3,980	27.5	2,398	39.8	1,904	20.3	1,385	35.4
1-4	7,855	54.3	2,744	45.6	5,282	56.2	1,814	46.4
≥5	1,808	12.5	496	8.2	1,864	19.8	521	13.3
Unknown	816	5.6	382	6.4	353	3.8	191	4.9
EDUCATION (YEARS^*^)								
< 10	3,640	25.2	2,574	42.8	1,344	14.3	1,305	33.4
10-12	5,714	39.5	2,196	36.5	2,847	30.3	1,288	32.9
≥13	4,817	34.0	1,111	18.5	5,117	54.4	1,248	31.9
Unknown	188	1.3	139	2.3	95	1.0	70	1.8
**ECONOMIC DIFFICULTIES**								
Yes	4,295	29.7	1,843	30.6	2,474	26.3	1,057	27.0
No	8,964	62.0	3,266	54.3	6,453	68.6	2,187	55.9
Unknown	1,200	8.3	911	15.1	476	5.1	667	17.1
**FAMILY HISTORY OF CANCER**								
Yes	3,944	27.3	1,801	29.9	2,446	26.0	1,166	29.8
No	10,515	72.7	4,219	70.1	6,957	74.0	2,745	70.2
**CHRONIC BRONCHITIS**								
Yes	402	2.8	229	3.8	234	2.5	169	4.3
No	13,861	95.7	5,677	94.3	9,067	96.4	3,678	94.0
Unknown	196	1.4	114	1.9	102	1.1	64	1.6
**OCCUPATIONAL ACTIVITY**								
Most sedentary work	1,389	9.6	668	11.1	5,322	56.6	1,959	50.1
Much walking or lifting at work	8,966	62.0	3,385	56.2	2,650	28.2	938	24.0
Heavy physical work	2,225	15.4	771	12.8	397	4.2	159	4.0
Unknown	1,879	13.0	1,196	19.9	1,034	11.0	855	21.9

*Continuous variables are presented with mean and standard deviation. Categorical variables are presented with number and column percentage of observations. Physically active: physical activity level from low to high. Physically inactive: reported no activity or only light activity ≤2 h per week*.

* *Years of education were reported as a positive integer*.

In Table 3, categories of total sitting time daily were not associated with lung cancer risk overall, SCLC or NSCLC in the main cohort after adjustment for a number of potential confounding factors including smoking status and physical activity. Total sitting time classified by tertiles was not associated with lung cancer risk either (data not presented). Results in ever smokers were similar to the main cohort (Supplementary Table 2). We were not able to perform analysis in never smokers as there were only 26 cases of lung cancer overall, no cases of SCLC and 19 cases of NSCLC among the never smokers.

**Table 3:** The association of total sitting time with lung cancer risk overall and histologic types, the HUNT Study, 1995–97 to 2014 (*N* = 45,810).

		**Cases**	**IR (per 1000 person-years)**	**Crude HR**	**95% CI**	**Adjusted^a^ HR**	**95% CI**
LC overall	Sitting 0–4 h	185	0.76	1.00	Reference	1.00	Reference
	Sitting 5–7 h	165	0.67	0.86	0.70–1.06	0.82	0.66–1.01
	Sitting ≥8 h	199	0.69	1.09	0.89–1.33	1.05	0.66–1.29
SCLC	Sitting 0–4 h	25	0.10	1.00	Reference	1.00	Reference
	Sitting 5–7 h	20	0.08	0.78	0.43–1.40	0.73	0.40–1.31
	Sitting ≥8 h	31	0.11	1.28	0.75–2.17	1.18	0.69–2.03
NSCLC	Sitting 0–4 h	117	0.48	1.00	Reference	1.00	Reference
	Sitting 5–7 h	97	0.40	0.80	0.61–1.05	0.76	0.58–1.00
	Sitting ≥8 h	119	0.41	1.02	0.79–1.32	0.97	0.74–1.26

*CI, Confidence interval; HR, Hazard ratio; IR, Incidence rate; LC, Lung cancer; NSCLC, Non-small cell lung cancer; SCLC, Small cell lung cancer*.

a *Adjusted for sex, body mass index, smoking status (pack-years), passive smoking status, physical activity, alcohol consumption, education, economic difficulties, family history of cancer and chronic bronchitis. Age is used as the time scale*.

Table 4 presents the association of the combined groups of total sitting time and physical activity with lung cancer risk overall and different histologic types. Compared to participants sitting < 8 h and being physically active, participants sitting ≥8 h and being physically inactive had increased risks of lung cancer overall and SCLC in the main cohort (overall: adjusted HR = 1.44, 95% CI: 1.07–1.94; SCLC: adjusted HR = 2.58, 95% CI: 1.23–5.41). Neither of the group with prolonged sitting only or physical inactivity only was associated with lung cancer risk. Similar results were found among ever smokers (Supplementary Table 3).

**Table 4:** The association of combined groups of total sitting time and physical activity with lung cancer risk overall and different histologic types, the HUNT Study, 1995–97 to 2014 (*N* = 33,793).

		**Cases**	**IR (per 1,000 person-years)**	**Crude HR**	**95% CI**	**Adjusted^a^ HR**	**95% CI**
LC overall	Sitting < 8 h & Physically active^b^	133	0.52	1.00	Reference	1.00	Reference
	Sitting < 8 h & Physically inactive^c^	81	0.80	1.15	0.87–1.52	1.06	0.80–1.40
	Sitting ≥8 h & Physically active^b^	62	0.37	0.87	0.64–1.18	0.93	0.68–1.26
	Sitting ≥8 h & Physically inactive^c^	70	1.11	1.74	1.30–2.32	1.44	1.07–1.94
SCLC	Sitting < 8 h & Physically active^b^	15	0.06	1.00	Reference	1.00	Reference
	Sitting < 8 h & Physically inactive^c^	11	0.11	1.44	0.66–3.14	1.35	0.61–2.99
	Sitting ≥8 h & Physically active^b^	5	0.03	0.60	0.22–1.65	0.59	0.21–1.64
	Sitting ≥8 h & Physically inactive^c^	14	0.22	3.26	1.57–6.76	2.58	1.23–5.41
NSCLC	Sitting < 8 h & Physically active^b^	78	0.31	1.00	Reference	1.00	Reference
	Sitting < 8 h & Physically inactive^c^	54	0.54	1.34	0.95–1.90	1.21	0.85–1.72
	Sitting ≥8 h & Physically active^b^	41	0.25	0.96	0.65–1.40	1.01	0.68–1.48
	Sitting ≥8 h & Physically inactive^c^	39	0.62	1.68	1.15-2.48	1.36	0.92–2.01

*CI, Confidence interval; HR, Hazard ratio; IR, Incidence rate; LC, Lung cancer; NSCLC, Non-small cell lung cancer; SCLC, Small cell lung cancer*.

a *Adjusted for sex, body mass index, smoking status (pack-years), passive smoking status, alcohol consumption, education, economic difficulties, family history of cancer and chronic bronchitis. Age is used as the time scale*.

b *Physically active: physical activity level from low to high*.

c *Physically inactive: reported no activity or only light activity ≤2 h per week*.

In the sensitivity analysis, we found no association between occupational inactivity (mostly sedentary work) and lung cancer risk (Supplementary Table 4). The association of combined sitting ≥8 h and physical inactivity with lung cancer risk was not altered after excluding the first 3 years of follow-up (Supplementary Table 5). When grouping low level physical activity into the physical inactivity group (Supplementary Table 6), the associations of the combined sitting time ≥8 h and physical inactivity with the risks of lung cancer overall and histologic types became weaker compared to the original results. Complete case analysis for information of smoking showed comparable results for lung cancer overall (Supplementary Table 7).

## Discussion

### Main Findings

In this prospective cohort study with 549 incident lung cancer cases, total sitting time daily was not associated with lung cancer. However, compared with participants sitting < 8 h daily and being physically active, participants sitting ≥8 h daily and being physically inactive appeared to have increased risks of lung cancer overall and SCLC.

### Comparison With Previous Studies

Previous studies showed different results on the association between sedentary lifestyle and lung cancer risk (17–21, 23). Occupational sitting was shown to be either protective (17, 19) or not associated with lung cancer risk (18). Different adjustment for physical activity and education might explain the differences in the results. On the contrary, leisure-time TV watching was associated with an increased risk of lung cancer among Japanese men but not women (20). Residual confounding by smoking was likely to be the explanation. In addition, Japanese women seemed to be more active than men (4.5 h housework for women & 1 h for men daily), which might be another reason for the gender difference in lung cancer risk. However, no adjustment for physical activity was made in this study.

Total sitting time daily, as a better measure of sedentary lifestyle, was not associated with lung cancer risk in the current study. Our result was consistent with findings from the study by Wang et al. (23). We included both men and women and adjusted for levels of physical activity, whereas only menopausal women were included and no adjustment for physical activity was made in the referred study. In contrast, Lam et al. found a marginally increased risk of lung cancer associated with prolonged sitting among non-smokers (21). Although the cited study largely avoided confounding by smoking among non-smokers, the adjustment for physical activity only included vigorous activity. Leisure-time TV watching and occupational inactivity were also studied by Lam et al. but no associations with lung cancer risk were found. In our study, neither prolonged sitting nor occupational inactivity was independently associated with lung cancer incidence after adjustment for detailed categories of smoking and physical activity. However, we observed an increased risk of lung cancer among the most sedentary individuals who were both extendedly seated and physically inactive.

### Possible Mechanisms

Although the underlying mechanisms on how the most sedentary individuals might have an increased risk of lung cancer are unclear, animal studies showed that lack of activities might suppress lipoprotein lipase activity in skeletal muscles and reduce glucose uptake (5, 6). Both are related to metabolic disorder that have been shown to be risk factors for several malignancies (6, 36). In addition, some pre-clinical studies suggest that weight-bearing skeletal muscles are not highly engaged during inactivity (7–9). This may alter anti-cancer responses of myokines in the skeletal muscles and activate inflammatory pathways that are important for cancer development (7–9).

### Strengths and Limitations

Scientific evidence regarding total sitting time daily in relation to lung cancer risk overall and histologic types is scarce. Our study is the first prospective cohort study to investigate lung cancer risk among people who were both extendedly seated and physically inactive. In addition, the sample size of our study is relatively large with homogeneous study population and the follow-up period is long to allow for study of rare disease outcome such as lung cancer. The Cancer Registry of Norway records information about lung cancer diagnosis and different histologic types 1 year after the first diagnosis, and the information is soundly complete and accurate (26). The distribution of key baseline characteristics in both the main and sub-cohorts are generally similar to the original cancer-free population, suggesting no substantial selection bias. We also had information on a panel of potential confounders at baseline. In addition, we excluded participants with cancer at baseline in the main analysis and excluded the first 3 years of follow-up in the sensitivity analysis. Thus, potential reverse causation due to preexisting but undiagnosed lung cancer may not be a major problem.

However, our study has several limitations. First, misclassification of total sitting time and physical activity was possible due to self-reporting, and weak correlations with accelerometer counts (r≈0.3) were reported in a previous study (31). Since all information on exposures was collected at baseline before the diagnosis of lung cancer, it was more likely to be non-differential misclassification. We further used occupational inactivity as an alternative marker of sedentary lifestyle to test the robustness of the association between total sitting time and lung cancer risk, and similar results were observed. Additionally, in our sensitivity analysis, the magnitude of association of sitting time ≥8 h in combination with physical inactivity with the lung cancer risk was reduced by grouping individuals who had low level physical activity into the inactive group. This suggested that the original combination of prolonged sitting and physical inactivity identified the most sedentary individuals and thereby the highest risk group for lung cancer.

Second, individuals who were extendedly seated and physically inactive were more likely to be smokers than those who were shortly seated and physically active. To minimize confounding by smoking, we used detailed information on smoking status together with pack-years to categorize this variable, but we were not able to perform analysis in never smokers among which there were only 26 lung cancer cases. We further conducted complete case analysis for information on smoking and similar results were obtained. In addition, we redefined smoking status by including cessation years for former daily smokers and categorized subjects into the groups of never smokers, ex-smokers with smoking cessation >10.1 years, ex-smokers with smoking cessation ≤10.0 years, current smokers with 0–20.0 pack-years, and current smokers with >20.1 pack-years. The results were similar to our original findings (data not presented). Nevertheless, residual confounding by smoking cannot be excluded entirely. Other unmeasured factors such as hazardous occupational exposures might also confound the association. Indoor radon exposure is suggested to be the second important risk factor for lung cancer after smoking (37), but the level of indoor radon at any of the seven municipalities in Nord-Trøndelag was shown to be in the safety range (< 200 Bq/m3) (38). At last, we could not look into specific histologic types of NSCLC such as adenocarcinoma and squamous cell carcinoma due to limited statistical power.

In conclusion, our study suggested that prolonged sitting was not independently associated with lung cancer risk, but prolonged sitting in combination with physical inactivity might increase the risk of lung cancer. However, our results should be interpreted with caution due to a possibility of residual confounding of smoking.

## Ethics Statement

The study was carried out in accordance with the recommendation of the Regional Committee for Medical and Health Research Ethics of South-East Norway. All subjects gave written informed consent on participation in HUNT, linkage to previous HUNT surveys and specific registries in accordance with the Declaration of Helsinki. The protocol was approved by the Regional Committee for Medical and Health Research Ethics of South-East Norway.

## Author Contributions

Y-QS and X-MM contributed to the study design and statistical analyses. AL and X-MM were responsible for data collection. LJ conducted statistical analyses, interpreted and wrote the initial draft of the manuscript. All authors participated in the data interpretation, contributed to the manuscript writing with important intellectual content and approved the final version of the manuscript.

### Conflict of Interest Statement

The authors declare that the research was conducted in the absence of any commercial or financial relationships that could be construed as a potential conflict of interest.
