# Supplementary material for: Prolonged Sitting, Its Combination With Physical Inactivity and Incidence of Lung Cancer: Prospective Data From the HUNT Study
Source: Front Oncol. 2019 Feb 25;9:101. doi: 10.3389/fonc.2019.00101 (PMC6397867; doi:10.3389/fonc.2019.00101)
Supplement: Supplementary file 1 [file Data_Sheet_1.docx]

Supplementary table 1. Comparison of baseline characteristics of participants in the main and sub-cohorts with the original cancer-free population, the HUNT Study, 1995-97

| Variables | Original cancer-free population  (N = 59,070) | | Main Cohort  (N = 45,810) | | Sub-Cohort  (N = 33,793) | |
| --- | --- | --- | --- | --- | --- | --- |
| Age (years) | 48.7 | 16.8 | 48.1 | 16.4 | 45.2 | 15.7 |
| Body mass index (kg/m^2^) | 26.3 | 4.1 | 26.2 | 4.0 | 26.1 | 4.0 |
| Sex |  | | | | | |
| Female | 30,819 | 52.2 | 23,786 | 51.9 | 16,879 | 50.0 |
| Male | 28,251 | 47.8 | 22,024 | 48.1 | 16,914 | 50.0 |
| Smoking status (pack-years) |  | | | | | |
| Never | 25,299 | 42.8 | 19,733 | 43.1 | 15,121 | 44.8 |
| Former ≤10.0 | 7,826 | 13.3 | 6,434 | 14.0 | 5,098 | 15.1 |
| Former 10.1-20.0 | 3,116 | 5.3 | 2,569 | 5.6 | 1,814 | 5.4 |
| Former >20.1 | 1,911 | 3.2 | 1,543 | 3.4 | 1,038 | 3.1 |
| Current ≤10.1 | 6,366 | 10.8 | 4,827 | 10.5 | 3,814 | 11.3 |
| Current 10.1-20.0 | 5,638 | 9.5 | 4,231 | 9.2 | 2,944 | 8.7 |
| Current >20.1 | 4,087 | 6.9 | 3,114 | 6.8 | 2,078 | 6.2 |
| Unknown | 4,827 | 8.2 | 3,359 | 7.3 | 1,886 | 5.6 |
| Passive smoking status |  | | | | | |
| Never | 10,889 | 18.4 | 8,502 | 18.6 | 6,417 | 19.0 |
| Only childhood | 12,587 | 21.3 | 10,037 | 21.9 | 8,087 | 23.9 |
| Only adulthood | 9,539 | 16.2 | 7,277 | 15.9 | 4,873 | 14.4 |
| Both | 24,640 | 41.7 | 19,101 | 41.7 | 13,999 | 41.4 |
| Unknown | 1,415 | 2.4 | 893 | 2.0 | 417 | 1.2 |
| Alcohol consumption (times/month) |  | | | | | |
| Never | 19,910 | 33.7 | 14,728 | 32.2 | 9,667 | 28.6 |
| 1-4 | 27,663 | 46.8 | 22,314 | 48.7 | 17,695 | 52.4 |
| ≥5 | 6,907 | 11.7 | 5,653 | 12.3 | 4,689 | 13.9 |
| Unknown | 4,590 | 7.8 | 3,115 | 6.8 | 1,742 | 5.2 |
| Education (years) |  | | | | | |
| <10 | 19,652 | 33.3 | 14,378 | 31.4 | 8,863 | 26.2 |
| 10-12 | 19,672 | 33.3 | 15,695 | 34.3 | 12,045 | 35.6 |
| ≥13 | 17,428 | 29.5 | 14,405 | 31.5 | 12,393 | 36.7 |
| Unknown | 2,318 | 3.9 | 1,332 | 2.9 | 492 | 1.5 |
| Economic difficulties |  | | | | | |
| Yes | 12,974 | 22.0 | 12,334 | 26.9 | 9,669 | 28.6 |
| No | 29,065 | 49.2 | 27,298 | 59.6 | 20,870 | 61.8 |
| Unknown | 17,031 | 28.8 | 6,178 | 13.5 | 3,254 | 9.6 |
| Family history of cancer |  | | | | | |
| Yes | 14,757 | 25.0 | 13,441 | 29.3 | 9,357 | 27.7 |
| No | 44,313 | 75.0 | 32,369 | 70.7 | 24,436 | 72.3 |
| Chronic bronchitis |  | | | | | |
| Yes | 1,914 | 3.2 | 1,454 | 3.2 | 1,034 | 3.1 |
| No | 56,054 | 94.9 | 43,554 | 95.1 | 32,283 | 95.5 |
| Unknown | 1,102 | 1.9 | 802 | 1.8 | 476 | 1.4 |
| Occupational activity |  | | | | | |
| Most sedentary work | 13,835 | 23.5 | 11,281 | 24.6 | 9,338 | 27.6 |
| Much walking or lifting at  work | 25,722 | 43.5 | 20,410 | 44.5 | 15,939 | 47.2 |
| Heavy physical work | 5,911 | 10.0 | 4,602 | 10.1 | 3,552 | 10.5 |
| Unknown | 13,602 | 23.0 | 9,517 | 20.8 | 4,964 | 14.7 |

Abbreviations: HUNT, Nord-Trøndelag Health Study; SD, standard deviation. Continuous variables are presented with mean and standard deviation.Categorical variables are presented with number and column percentage of observations.

Supplementary table 2. The association of total sitting time with lung cancer risk overall and histologic types in ever smokers, the HUNT Study, 1995-97 to 2014 (N=25,449)

|  |  | N | Cases | IR  (per 1000 person-years) | Crude  HR | Adjusted^1^  HR | 95% CI |
| --- | --- | --- | --- | --- | --- | --- | --- |
| LC overall | Sitting 0-4 hours | 7,832 | 171 | 1.30 | 1.00 | 1.00 | Reference |
|  | Sitting 5-7 hours | 8,284 | 155 | 1.13 | 0.85 | 0.83 | 0.67-1.03 |
|  | Sitting ≥8 hours | 9,333 | 189 | 1.21 | 1.09 | 1.07 | 0.87-1.33 |
| SCLC | Sitting 0-4 hours | 7,832 | 24 | 0.18 | 1.00 | 1.00 | Reference |
|  | Sitting 5-7 hours | 8,284 | 20 | 0.15 | 0.78 | 0.75 | 0.42-1.37 |
|  | Sitting ≥8 hours | 9,333 | 31 | 0.20 | 1.27 | 1.23 | 0.71-2.14 |
| NSCLC | Sitting 0-4 hours | 7,832 | 108 | 0.82 | 1.00 | 1.00 | Reference |
|  | Sitting 5-7 hours | 8,284 | 90 | 0.65 | 0.78 | 0.76 | 0.58-1.01 |
|  | Sitting ≥8 hours | 9,333 | 111 | 0.71 | 1.00 | 0.97 | 0.74-1.27 |

Abbreviation: CI, Confidence interval; HR, Hazard ratio; HUNT, Nord-Trøndelag Health Study; IR, Incidence rate; LC, Lung cancer; NSCLC, Non-small cell lung cancer; SCLC, Small cell lung cancer. ^1^ Adjusted for sex, body mass index, smoking status (pack-years), passive smoking status, physical activity, alcohol consumption, education, economic difficulties, family history of cancer and chronic bronchitis. Age is used as the time scale.

Supplementary table 3. The association of combined groups of total sitting time and physical activity with lung cancer risk overall and different histologic types in ever smokers, the HUNT Study, 1995-97 to 2014 (N=18,335)

|  |  | N | Cases | IR  (per 1000 person-years) | Crude  HR | Adjusted^1^  HR | 95% CI |
| --- | --- | --- | --- | --- | --- | --- | --- |
| LC overall | Sitting <8 hours &Physically active^2^ | 7,668 | 118 | 0.89 | 1.00 | 1.00 | Reference |
|  | Sitting <8 hours &Physically inactive^3^ | 3,541 | 76 | 1.30 | 1.26 | 1.13 | 0.85-1.52 |
|  | Sitting ≥8 hours &Physically active^2^ | 4,764 | 58 | 0.70 | 0.91 | 0.99 | 0.71-1.36 |
|  | Sitting ≥8 hours &Physically inactive^3^ | 2,362 | 69 | 1.80 | 1.93 | 1.61 | 1.19-2.18 |
| SCLC | Sitting <8 hours &Physically active^2^ | 7,668 | 15 | 0.11 | 1.00 | 1.00 | Reference |
|  | Sitting <8 hours &Physically inactive^3^ | 3,541 | 10 | 0.17 | 1.30 | 1.22 | 0.54-2.77 |
|  | Sitting ≥8 hours &Physically active^2^ | 4,764 | 5 | 0.06 | 0.60 | 0.58 | 0.21-1.63 |
|  | Sitting ≥8 hours &Physically inactive^3^ | 2,362 | 14 | 0.37 | 3.06 | 2.55 | 1.22-5.36 |
| NSCLC | Sitting <8 hours &Physically active^2^ | 7,668 | 67 | 0.51 | 1.00 | 1.00 | Reference |
|  | Sitting <8 hours &Physically inactive^3^ | 3,541 | 51 | 0.87 | 1.49 | 1.34 | 0.92-1.94 |
|  | Sitting ≥8 hours &Physically active^2^ | 4,764 | 38 | 0.46 | 1.03 | 1.09 | 0.73-1.64 |
|  | Sitting ≥8 hours &Physically inactive^3^ | 2,362 | 39 | 1.02 | 1.91 | 1.58 | 1.05-2.36 |

Abbreviation: CI, Confidence interval; HR, Hazard ratio; HUNT, Nord-Trøndelag Health Study; IR, Incidence rate; LC, Lung cancer; NSCLC, Non-small cell lung cancer; SCLC, Small cell lung cancer. ^1^ Adjusted for sex, body mass index, smoking status (pack-years), passive smoking status, alcohol consumption, education, economic difficulties, family history of cancer and chronic bronchitis Age is used as the time scale.^2^ Physically active: physical activity level from low to high. ^3^ Physically inactive: reported no activity or only light activity ≤2 hours per week.

Supplementary table 4. The association of occupational inactivity with lung cancer risk overall and histologic types, the HUNT Study, 1995-97 to 2014 (N=45,810)

|  |  | N | Cases | IR  (per 1000 person-years) | Crude  HR | Adjusted^1^  HR | 95% CI |
| --- | --- | --- | --- | --- | --- | --- | --- |
| LC overall | Much walking or lifting at work | 20,410 | 189 | 0.52 | 1.00 | 1.00 | Reference |
|  | Most sedentary work | 11,281 | 102 | 0.52 | 0.92 | 0.97 | 0.75-1.24 |
|  | Heavy physical work | 4,602 | 47 | 0.58 | 0.98 | 0.79 | 0.57-1.10 |
|  | Unknown | 9,517 | 211 | 1.55 | 1.13 | 1.09 | 0.87-1.36 |
| SCLC | Much walking or lifting at work | 20,410 | 28 | 0.08 | 1.00 | 1.00 | Reference |
|  | Most sedentary work | 11,281 | 13 | 0.07 | 0.81 | 0.79 | 0.40-1.55 |
|  | Heavy physical work | 4,602 | 2 | 0.02 | 0.28 | 0.23 | 0.05-0.98 |
|  | Unknown | 9,517 | 33 | 0.24 | 1.45 | 1.35 | 0.77-2.37 |
| NSCLC | Much walking or lifting at work | 20,410 | 121 | 0.33 | 1.00 | 1.00 | Reference |
|  | Most sedentary work | 11,281 | 61 | 0.31 | 0.87 | 0.90 | 0.66-1.23 |
|  | Heavy physical work | 4,602 | 33 | 0.41 | 1.08 | 0.87 | 0.58-1.29 |
|  | Unknown | 9,517 | 118 | 0.87 | 1.07 | 1.02 | 0.77-1.36 |

Abbreviation: CI, Confidence interval; HR, Hazard ratio; HUNT, Nord-Trøndelag Health Study; IR, Incidence rate; LC, Lung cancer; NSCLC, Non-small cell lung cancer; SCLC, Small cell lung cancer. ^1^ Adjusted for sex, body mass index, smoking status (pack-years), passive smoking status, physical activity, alcohol consumption, education, economic difficulties, family history of cancer and chronic bronchitis. Age is used as the time scale.

Supplementary table 5. The association of combined groups of total sitting time and physical activity with lung cancer risk overall and different histologic types after excluding the first three years’ follow-up for all participants, the HUNT Study, 1995-97 to 2014 (N = 33,322)

|  |  | N | Cases | IR  (per 1000 person-years) | Crude HR | Adjusted^1^  HR | 95% CI |
| --- | --- | --- | --- | --- | --- | --- | --- |
| LC overall | Sitting <8 hours & Physically active^2^ | 14,321 | 117 | 0.56 | 1.00 | 1.00 | Reference |
|  | Sitting <8 hours & Physically inactive^3^ | 5,898 | 74 | 0.89 | 1.22 | 1.09 | 0.82-1.47 |
|  | Sitting ≥8 hours & Physically active^2^ | 9,322 | 52 | 0.38 | 0.82 | 0.87 | 0.63-1.22 |
|  | Sitting ≥8 hours & Physically inactive^3^ | 3,781 | 62 | 1.20 | 1.82 | 1.47 | 1.07-2.00 |
| SCLC | Sitting <8 hours & Physically active^2^ | 14,321 | 15 | 0.07 | 1.00 | 1.00 | Reference |
|  | Sitting <8 hours & Physically inactive^3^ | 5,898 | 10 | 0.12 | 1.34 | 1.22 | 0.54-2.76 |
|  | Sitting ≥8 hours & Physically active^2^ | 9,322 | 4 | 0.03 | 0.47 | 0.45 | 0.54-2.76 |
|  | Sitting ≥8 hours & Physically inactive^3^ | 3,781 | 13 | 0.25 | 3.13 | 2.38 | 1.12-5.08 |
| NSCLC | Sitting <8 hours & Physically active^2^ | 14,321 | 68 | 0.32 | 1.00 | 1.00 | Reference |
|  | Sitting <8 hours & Physically inactive^3^ | 5,898 | 48 | 0.58 | 1.39 | 1.21 | 0.83-1.77 |
|  | Sitting ≥8 hours & Physically active^2^ | 9,322 | 36 | 0.26 | 0.95 | 0.99 | 0.66-1.50 |
|  | Sitting ≥8 hours & Physically inactive^3^ | 3,781 | 34 | 0.66 | 1.74 | 1.36 | 0.90-2.07 |

Abbreviation: CI, Confidence interval; HR, Hazard ratio; HUNT, Nord-Trøndelag Health Study; IR, Incidence rate; LC, Lung cancer; NSCLC, Non-small cell lung cancer; SCLC, Small cell lung cancer. ^1^ Adjusted for sex, body mass index, smoking status (pack-years), passive smoking status, alcohol consumption, education, economic difficulties, family history of cancer and chronic bronchitis. Age is used as the time scale.^2^ physically active: physical activity level from low to high. ^3^ Physically inactive: reported no activity or only light activity ≤2 hours per week.

Supplementary table 6. The association of combined groups of total sitting time and physical activity with lung cancer risk overall and different histologic types after including low activity into the physically inactive group, the HUNT Study, 1995-97 to 2014 (N = 33,793)

|  |  | N | Cases | IR  (per 1000 person-years) | Crude HR | Adjusted^1^  HR | 95% CI |
| --- | --- | --- | --- | --- | --- | --- | --- |
| LC overall | Sitting <8 hours & Physically active^2^ | 9,320 | 70 | 0.42 | 1.00 | 1.00 | Reference |
|  | Sitting <8 hours & Physically inactive^3^ | 11,159 | 144 | 0.76 | 1.27 | 1.16 | 0.87-1.55 |
|  | Sitting ≥8 hours & Physically active^2^ | 6,072 | 30 | 0.28 | 0.79 | 0.89 | 0.58-1.37 |
|  | Sitting ≥8 hours & Physically inactive^3^ | 7,242 | 102 | 0.84 | 1.63 | 1.40 | 1.03-1.90 |
| SCLC | Sitting <8 hours & Physically active^2^ | 9,320 | 8 | 0.05 | 1.00 | 1.00 | Reference |
|  | Sitting <8 hours & Physically inactive^3^ | 11,159 | 18 | 0.10 | 1.44 | 1.34 | 0.57-3.12 |
|  | Sitting ≥8 hours & Physically active^2^ | 6,072 | 2 | 0.02 | 0.44 | 0.47 | 0.10-2.24 |
|  | Sitting ≥8 hours & Physically inactive^3^ | 7,242 | 17 | 0.14 | 2.47 | 1.99 | 0.85-4.67 |
| NSCLC | Sitting <8 hours & Physically active^2^ | 9,320 | 43 | 0.26 | 1.00 | 1.00 | Reference |
|  | Sitting <8 hours & Physically inactive^3^ | 11,159 | 89 | 0.47 | 1.31 | 1.17 | 0.81-1.70 |
|  | Sitting ≥8 hours & Physically active^2^ | 6,072 | 22 | 0.20 | 0.92 | 1.01 | 0.60-1.71 |
|  | Sitting ≥8 hours & Physically inactive^3^ | 7,242 | 58 | 0.48 | 1.53 | 1.27 | 0.85-1.90 |

Abbreviation: CI, Confidence interval; HR, Hazard ratio; HUNT, Nord-Trøndelag Health Study; IR, Incidence rate; LC, Lung cancer; NSCLC, Non-small cell lung cancer; SCLC, Small cell lung cancer. ^1^ Adjusted for sex, body mass index, smoking status (pack-years), passive smoking status, alcohol consumption, education, economic difficulties, family history of cancer and chronic bronchitis. Age is used as the time scale. ^2^ Physically active: physical activity level from moderate to high. ^3^ Physically inactive: reported no activity or any light activity only or light activity ≤2 hours and hard activity <1 hour per week.

Supplementary table 7. Complete case analysis regarding information on smoking status for the association of combined groups of total sitting time and physical activity with lung cancer risk overall and different histologic types, the HUNT Study, 1995-97 to 2014 (N=31,907)

|  |  | N | Cases | IR  (per 1000 person-years) | Crude  HR | Adjusted^1^  HR | 95% CI |
| --- | --- | --- | --- | --- | --- | --- | --- |
| LC overall | Sitting <8 hours & Physically active^2^ | 13,605 | 116 | 0.48 | 1.00 | 1.00 | Reference |
|  | Sitting <8 hours & Physically inactive^3^ | 5,634 | 73 | 0.77 | 1.19 | 1.05 | 0.78-1.41 |
|  | Sitting ≥8 hours & Physically active^2^ | 8,977 | 58 | 0.36 | 0.90 | 0.97 | 0.70-1.33 |
|  | Sitting ≥8 hours & Physically inactive^3^ | 3,691 | 64 | 1.06 | 1.79 | 1.44 | 1.06-1.97 |
| SCLC | Sitting <8 hours & Physically active^2^ | 13,605 | 15 | 0.06 | 1.00 | 1.00 | Reference |
|  | Sitting <8 hours & Physically inactive^3^ | 5,634 | 10 | 0.11 | 1.30 | 1.21 | 0.53-2.75 |
|  | Sitting ≥8 hours & Physically active^2^ | 8,977 | 5 | 0.03 | 0.59 | 0.57 | 0.20-1.59 |
|  | Sitting ≥8 hours & Physically inactive^3^ | 3,691 | 10 | 0.17 | 2.27 | 1.72 | 0.77-3.88 |
| NSCLC | Sitting <8 hours & Physically active^2^ | 13,605 | 70 | 0.29 | 1.00 | 1.00 | Reference |
|  | Sitting <8 hours & Physically inactive^3^ | 5,634 | 47 | 0.50 | 1.30 | 1.14 | 0.78-1.66 |
|  | Sitting ≥8 hours & Physically active^2^ | 8,977 | 39 | 0.24 | 0.98 | 1.03 | 0.69-1.54 |
|  | Sitting ≥8 hours & Physically inactive^3^ | 3,691 | 37 | 0.62 | 1.75 | 1.40 | 0.94-2.10 |

Abbreviation: CI, Confidence interval; HR, Hazard ratio; HUNT, Nord-Trøndelag Health Study; IR, Incidence rate; LC, Lung cancer; NSCLC, Non-small cell lung cancer; SCLC, Small cell lung cancer. ^1^ Adjusted for sex, body mass index, smoking status (pack-years), passive smoking status, alcohol consumption, education, economic difficulties, family history of cancer and chronic bronchitis. Age is used as the time scale.^2^ Physically active: physical activity level from low to high. ^3^ Physically inactive: reported no activity or only light activity ≤2 hours per week.
